# Association Between Cerebellar Metabolic Markers and Activities of Daily Living in Patients With Spinocerebellar Ataxia Type 3

**DOI:** 10.1002/mgg3.70197

**Published:** 2026-04-15

**Authors:** Mei Ye, Xiao Ping Qiu, Shengnan Zhang, Ling Wang, Zhongming Gao, Mengyu Liu, Yaping Feng

**Affiliations:** ^1^ Department of Neurology The Affiliated Hospital of Hangzhou Normal University Hangzhou China

**Keywords:** activities of daily living, MRS, neuroimaging biomarkers, spinocerebellar ataxia type 3

## Abstract

**Background:**

Spinocerebellar ataxia type 3 (SCA3) is a progressive neurodegenerative disorder that results in the impaired ability to perform activities of daily living (ADLs). However, there is a lack of objective neuroimaging indices to assess functional decline. In this study, we investigated the association between cerebellar metabolic rate as detected by magnetic resonance spectroscopy (MRS) and ADL performance in patients with SCA3.

**Methods:**

A total of 23 SCA3 patients underwent multivoxel MRS scans of the cerebellar cortex, dentate nucleus, and vermis and were analyzed for associations between the metabolic ratio and the ability to perform activities of daily living (ADL). Metabolic ratios were quantified and correlated with Barthel Index scores. The predictive value of metabolic ratio was assessed using multiple regression and least absolute shrinkage and selection operator (LASSO) analysis.

**Results:**

Our results showed that the NAA/Cr and Cho/Cr ratios in all three brain regions were significantly correlated with higher Barthel Index scores (*r* = 0.66), indicating a link between neuronal integrity, membrane metabolism, and functional independence. Regression analyses confirmed these associations, and the exploratory LASSO analysis suggested that the combined markers may have potential predictive value in patients' daily lives.

**Conclusion:**

Cerebellar NAA/Cr and Cho/Cr ratios may serve as candidate neuroimaging biomarkers of functional status in patients with SCA3. These preliminary results warrant validation in future studies using advanced MRS quantitative analysis.

## Graphical abstract

Significant correlations were found between cerebellar metabolite ratios and Activities of Daily Living (ADL) in SCA3 patients. These findings suggest that cerebellar metabolite ratios may serve as potential neuroimaging biomarkers to predict functional disability in SCA3.

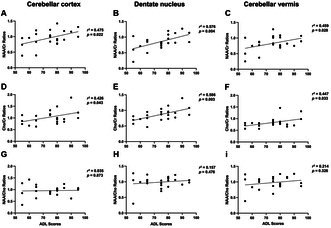

## 1 Introduction

Spinocerebellar ataxia type 3 (SCA3, also known as Machado‐Joseph disease) is an autosomal dominant neurodegenerative disease defined by Cytosin‐Adenin‐Guanin (CAG) trinucleotide repeat expansions in the *ATXN3* gene (OMIM: 607047) (Kawaguchi et al. 1994), and it is marked by clinical features of progressive cerebellar ataxia, extrapyramidal signs, oculomotor abnormalities, hypotonia, and peripheral neuropathy (Bettencourt and Lima 2011). As the disease progresses, patients progressively lose the capacity to accomplish activities of daily living (ADLs) (Chen, Tian, et al. 2025; Potashman et al. 2025). At present, repetitive transcranial magnetic stimulation (rTMS) offers a noninvasive therapeutic approach that may partially improve ataxia symptoms, yet no effective disease‐modifying treatment can reverse disease progression (Klockgether et al. 2019; Chen, Zhou, et al. 2025). Thus, the early detection of SCA3 patients with a high risk of functional deterioration and the timely implementation of effective, targeted interventions play primary roles in stalling the disease.

Magnetic resonance spectroscopy (MRS) noninvasively quantifies brain metabolites (Wang et al. 2012). Of these, N‐acetyl aspartate (NAA) is considered a neuronal viability marker, and its ratio to creatine (NAA/Cr) is widely used to evaluate neuronal damage (Peng et al. 2019). Choline (Cho) reflects the cell membrane turnover and gliosis, for which Cho/Cr presents the glial pathology and membrane metabolism (Chen et al. 2014). In previous studies, a significant decrease in the NAA/Cr ratio was detected in SCA3 patients that was negatively correlated with the scale for the assessment and rating of ataxia (SARA) scores (Chen et al. 2024; Chandrasekaran et al. 2023), which suggests a potential role for brain metabolites in evaluating the ataxia severity in SCA3. While associations between brain metabolites and ataxia severity in SCA3 have been established, the link between these metabolites and functional capacities in daily life remains elusive (Krahe et al. 2020; Wang et al. 2012; Wan et al. 2020).

Activities of Daily Living scales are used to evaluate SCA3 patients' ability to manage fundamental self‐care activities, encompassing domains such as feeding, grooming, and mobility. Since cerebral metabolic abnormalities often precede the onset of clinical symptoms (Peng et al. 2019), linking brain metabolites to ADL scores can help predict future functional deterioration and guide early intervention.

In this study, we analyzed the correlation between MRS‐detected cerebellar metabolic ratios and ADL (Barthel Index scores) in SCA3 patients. We also performed multivariable models to correct for confounding variables, such as the severity of ataxia (SARA score), age, and age of onset, to evaluate the prognostic role of MRS‐detected metabolic markers with respect to patients' functional status. These cerebellar metabolic ratios could thus enable early functional surveillance and guide personalized care strategies in SCA3.

## 2 Methods

### 2.1 Participants

The study was approved by the Ethics Committee of Hangzhou Normal University Affiliated Hospital and written informed consent was obtained from all participants. Twenty‐three patients with SCA3 were recruited from the Department of Neurology of Hangzhou Normal University Affiliated Hospital. All of the patients have been genetically confirmed with expanded CAG repeats of the *ATXN3* gene (GenBank accession number NM_004993.6). We included patients (1) with a definite diagnosis of SCA3, (2) with full clinical and imaging data, and (3) who had been given informed consent. (4) age ≥ 18. Patients with other forms of ataxia or with other neurological comorbidities that influenced the cerebellar function were excluded. The clinical information of SCA3 patients was recorded, which included age, gender, age at onset of SCA3, disease duration, SARA score, ICARS score, and Barthel Index scores.

### 2.2 Imaging Acquisition and Processing

Cerebellar ^1^H‐MRS was performed using a Siemens 1.5 Tesla MRI scanner. Multivoxel MRS sequences were used to acquire metabolic spectra with the following parameters: repetition time (RT) = 4280 ms and echo time (ET) = 135 ms. Voxels (~6.3 × 6.3 × 15 mm) were placed over the three regions of interest (ROIs) in the cerebellum: the cerebellar cortex, the dentate nucleus, and the vermis. Peaks of N‐acetyl aspartate (NAA), creatine (Cr), and choline (Cho) were quantified for each ROI, and the ratios of NAA/Cr, Cho/Cr, and NAA/Cho were calculated by integrating the respective peak areas. MRS image processing and metabolite quantification were independently performed by two experienced radiologists who were blinded to the clinical data. For analysis, the average of the two raters' values was used, and no formal inter‐rater reliability testing was performed.

### 2.3 Assessment of ADL Function

The Barthel Index was applied to assess the daily functional status of the patients by scoring 10 activities (feeding, bathing, grooming, dressing, bowels, bladder, toilet use, transfers, mobility, and stairs) on a scale from 0 to 100. To quantify the level of ataxia, we used the SARA scale and the ICARS scale. Higher SARA and ICARS scores indicated more severe ataxia, whereas higher Barthel Index scores indicated greater independence.

### 2.4 Statistical Analyses

All analyses were done using the SPSS software (Version 29.0.2). Normality was tested for all variables by applying the Shapiro–Wilk test. Pearson's correlation analysis was applied to assess the association between MRS‐detected metabolic ratios (i.e., NAA/Cr, Cho/Cr, and NAA/Cho), the SARA and ICARS scores, and the Barthel Index scores. Spearman's rank correlation analysis was applied to compare the distribution of the metabolic ratios and the Barthel Index scores for non‐normal distributions. Additionally, multiple linear regression was performed to investigate the independent associations between the MRS‐detected metabolic markers and ADL performance, with the Barthel Index score as the dependent variable and the independent variables being the metabolic ratios. The confounders potential as the SARA score and age at onset, were included in the model for adjustment. Least absolute shrinkage and selection operator (LASSO) was performed to enhance the regression analysis between the metabolic changes and Barthel Index. A two‐tailed *p* value < 0.05 was regarded as statistically significant.

## 3 Results

### 3.1 Ataxia Severity and Functional Independence

**FIGURE 1:**
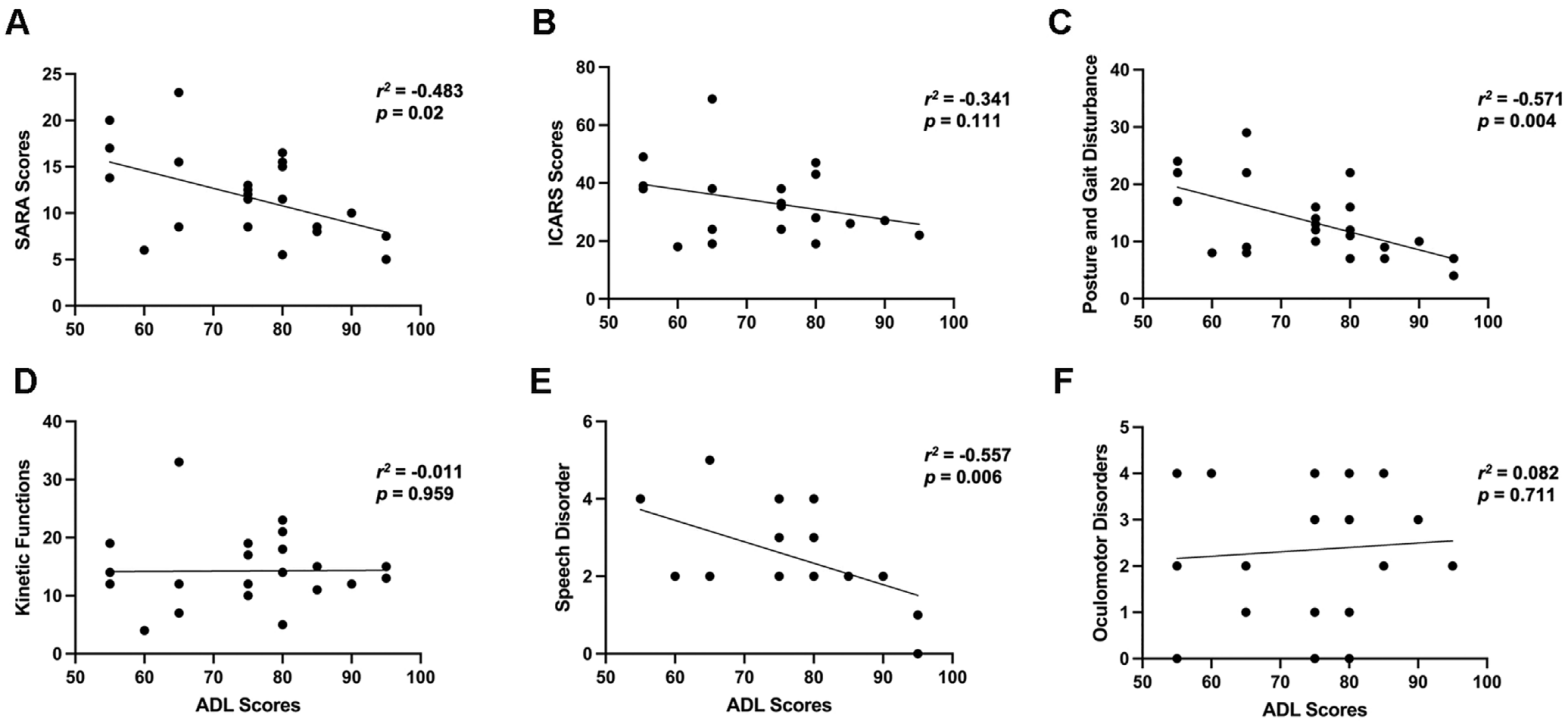
Correlations between ADL and clinical ataxia rating scales. (A) ADL were significantly negatively correlated with SARA scores. (B) A non‐significant negative correlation was observed with ICARS scores. (C) ADL were significantly negatively correlated with Posture and Gait Disturbance scores. (D) No significant correlation was found with Kinetic Functions. (E) A significant negative correlation was observed with Speech Disorder scores. (F) No significant correlation was found with Oculomotor Disorders. Pearson correlation coefficients (*r* ^2^) and *p*‐values are shown.

A total of twenty‐three SCA3 patients were enrolled. SARA scores are negatively correlated with ADL (*r* = −0.483, *p* = 0.02), indicating that more ataxia severity is associated with lower functional independence. The ICARS scores showed a negative correlation with ADLs (*r* ^2^ = −0.341), but without a significant *p*‐value (*p* = 0.111). Correlation analysis also shows for the separate ICARS subdomains that a lower posture and gait disturbance (*r* ^2^ = −0.571, *p* = 0.004) is significantly correlated with better functional independence. Impairment in limb kinetic dysfunction (*r* ^2^ = −0.011, *p* = 0.959), oculomotor abnormalities (*r* ^2^ = 0.082, *p* = 0.711) were not significantly associated with the ADL scores, whereas impairment speech disturbance (*r* ^2^ = −0.557, *p* = 0.006) was negatively significantly associated (Table 1, Figure 1).

**TABLE 1:** Correlations between ADL scores and clinical variables in SCA3 patients.

Variable	Mean ± Std	ADL Scores	
		*r* ^2^	*p*
Number	23		
Age (year)	40.39 ± 11.11	−0.008	0.97
Disease duration (year)	6.78 ± 3.01	−0.136	0.536
Age at onset (year)	33.48 ± 10.89	0.018	0.934
CAG repeat	74.22 ± 4.78	0.11	0.618
SARA scores	11.86 ± 4.69	−0.483	0.02*
ICARS scores	32.87 ± 12.14	−0.341	0.111
Posture and gait disturbance	13.43 ± 6.56	−0.571	0.004**
Kinetic functions	14.26 ± 6.25	0.011	0.959
Speech disorder	2.65 ± 1.19	−0.557	0.006**
Oculomotor disorders	2.35 ± 1.4	0.082	0.711
ADL scores	74.35 ± 11.99	N.A.	N.A.

Abbreviations: CAG repeat, Cytosine‐Adenine‐Guanine repeat in *Ataxin‐3* gene; ICARS Scores, International Cooperative Ataxia Rating Scale; SARA Scores, Scale for the Assessment and Rating of Ataxia.

Statistical significance: *p < 0.05, **p < 0.01.

Further, there were no significant correlations between ADL scores and age (*r* ^2^ = −0.008, *p* = 0.97), disease duration (*r* ^2^ = −0.136, *p* = 0.536), age at onset (*r* ^2^ = 0.018, *p* = 0.934), CAG repeat length (*r* ^2^ = 0.11, *p* = 0.618), limb function (*r* ^2^ = 0.011, *p* = 0.959), or oculomotor function (*r* ^2^ = 0.082, *p* = 0.711) (Table 1).

### 3.2 Biological Interpretation of the Metabolic Markers

To better understand the correlation between the cerebellar metabolism and the severity of ataxia, we performed a correlation analysis of the SARA scores with the cerebellar metabolic ratios (NAA/Cr, Cho/Cr, and NAA/Cho) in the three cerebellar regions. To contextualize our findings, we compared our results with two previously published studies that included both SCA3 patients and healthy controls (Table S1) (Chen et al. 2024; Peng et al. 2019). The metabolite ratios in our SCA3 cohort were consistent with previous SCA3 data and relatively lower than those of the healthy controls.

**FIGURE 2:**
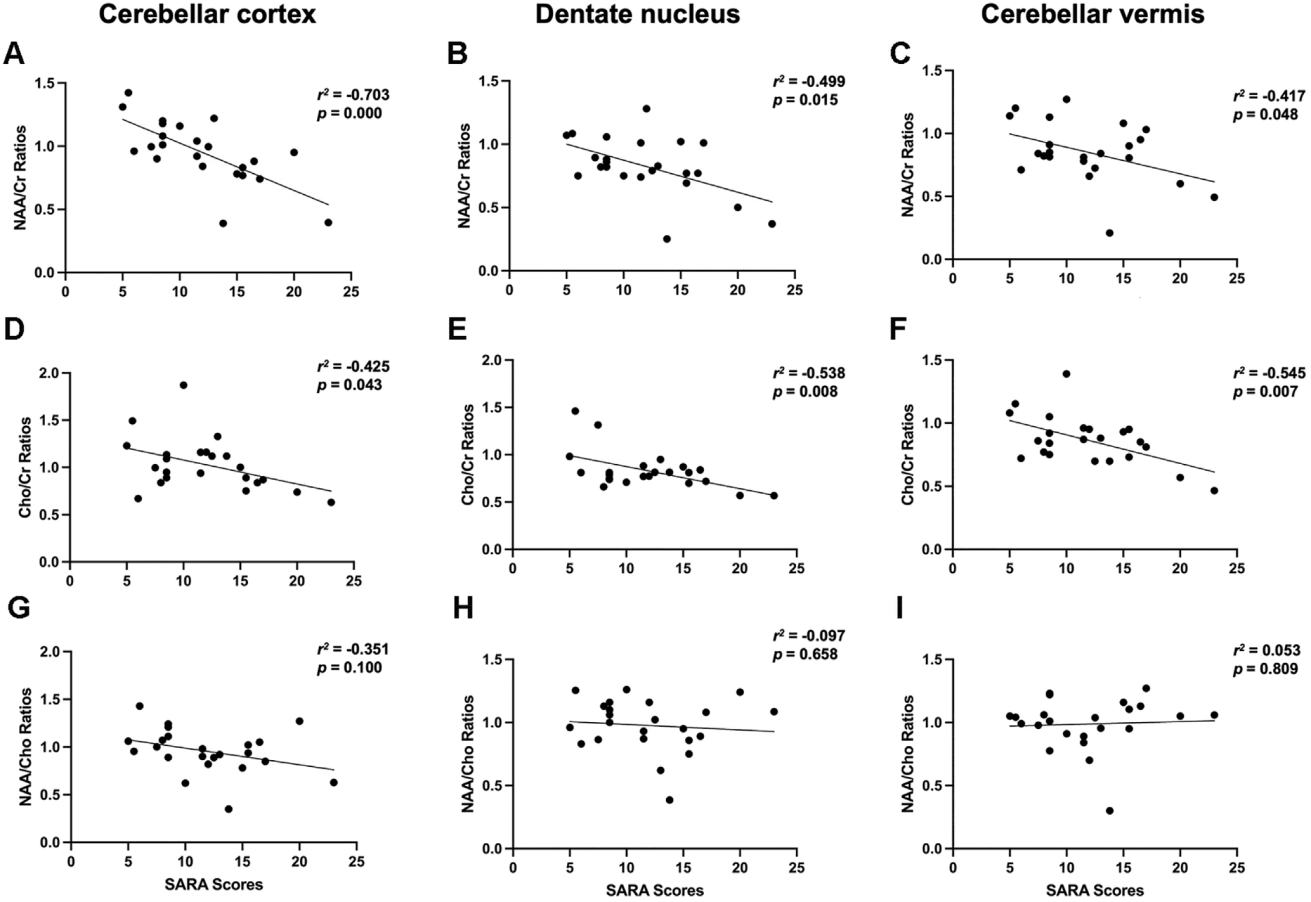
Correlations between brain metabolite ratios and SARA scores. In the cerebellar cortex, dentate nucleus, and cerebellar vermis: (A–C) NAA/Cr ratios showed significant negative correlations with SARA scores. (D–F) Cho/Cr ratios also showed significant negative correlations with SARA scores. (G–I) NAA/Cho ratios were not significantly correlated with SARA scores. Pearson correlation coefficients (*r* ^2^) and *p*‐values are shown.

As shown in Figure 2, the NAA/Cr ratios were significantly negatively correlated with the SARA scores in the cerebellar cortex (*r* ^2^ = −0.703, *p* < 0.001), the cerebellar dentate nucleus (*r* ^2^ = −0.499, *p* = 0.015), and the vermis (*r* ^2^ = −0.417, *p* = 0.007). These relationships implied lower neuronal integrity closely associated with the ataxia severity of SCA3. Likewise, the Cho/Cr ratios were negatively correlated with the SARA scores in the cortex (*r* ^2^ = −0.425, *p* = 0.043), in the dentate nucleus (*r* ^2^ = −0.538, *p* = 0.008), and in the vermis (*r* ^2^ = −0.545, *p* = 0.007), which suggested a relationship that indicated a possibly similar connection between disturbed membrane metabolism and disease (disease severity). Conversely, the NAA/Cho ratios showed no correlation in the cortex (*r* ^2^ = −0.351, *p* = 0.100), the dentate nucleus (*r* ^2^ = −0.097, *p* = 0.658), or the vermis (*r* ^2^ = 0.053, *p* = 0.809).

**FIGURE 3:**
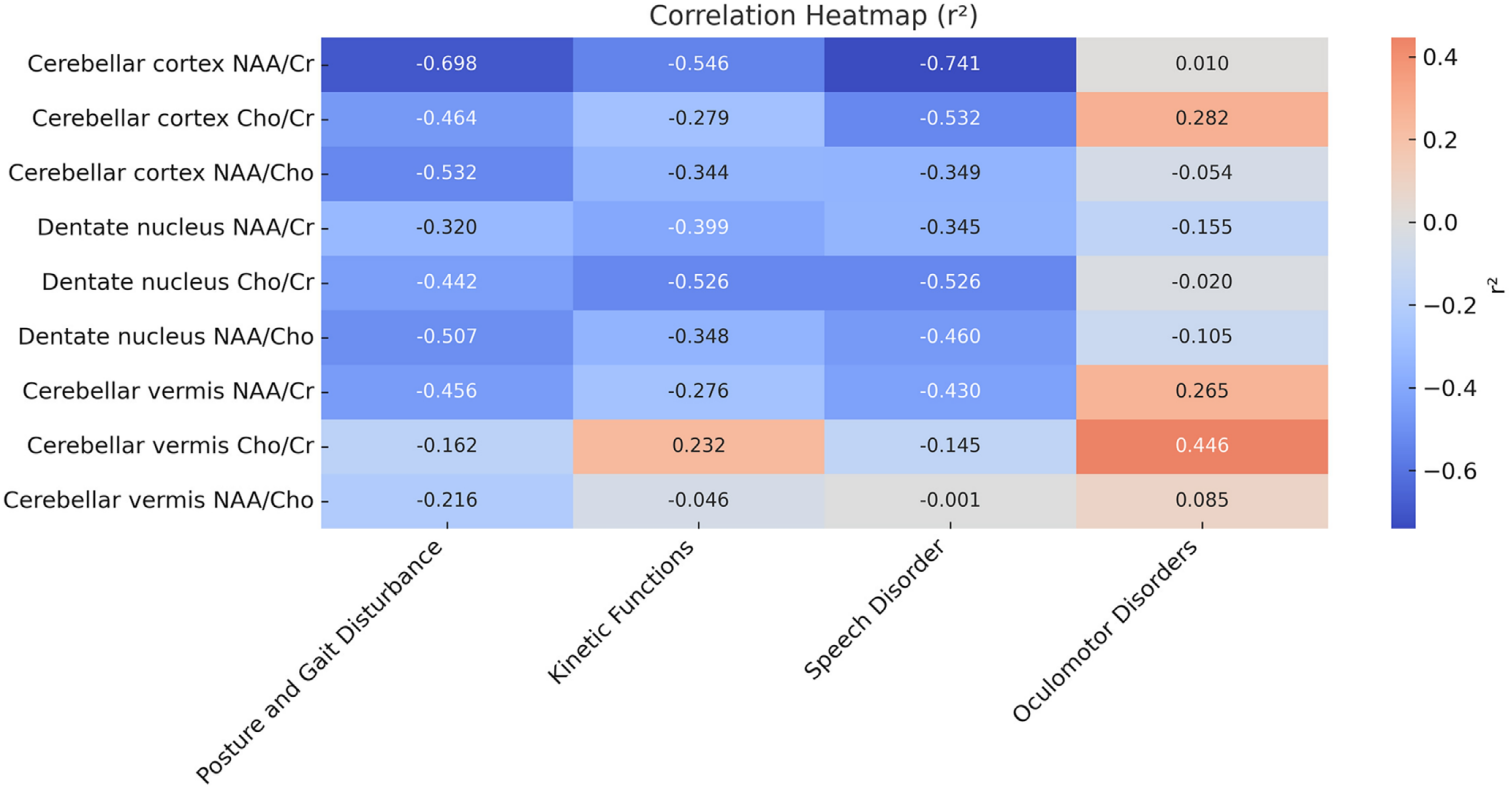
Correlation heatmap (*r* ^2^) of brain metabolite ratios with subdomains of ataxia. The heatmap displays the strength of correlations (*r* ^2^ values) between brain metabolite ratios (NAA/Cr, Cho/Cr, NAA/Cho) in three cerebellar regions and scores for Posture and Gait Disturbance, Kinetic Functions, Speech Disorder, and Oculomotor Disorders. Blue indicates negative correlations, red indicates positive correlations, with intensity reflecting the strength of correlation. *r* ^2^ values are shown.

To explore the associations between cerebellar metabolism and the clinical subdomains of ataxia, we generated a heatmap that indicated the correlation coefficients (*r* ^2^) between the metabolite ratios and the scores for gait and posture, limb coordination, speech, and oculomotor function (Figure 3). The NAA/Cr ratios in the cerebellar cortex were strongly negatively correlated with gait and posture disturbance (*r* ^2^ = −0.698), speech disturbance (*r* ^2^ = −0.741), and limb dysfunction (*r* ^2^ = −0.546), thereby indicating a close link between neuronal loss and motor impairment. Moreover, the Cho/Cr ratios in the dentate nucleus were also negatively correlated with limb dysfunction and speech disturbance (both *r* ^2^ = −0.526). Overall, the NAA/Cho ratios exhibited weak and mostly nonsignificant correlations across these clinical domains.

**FIGURE 4:**
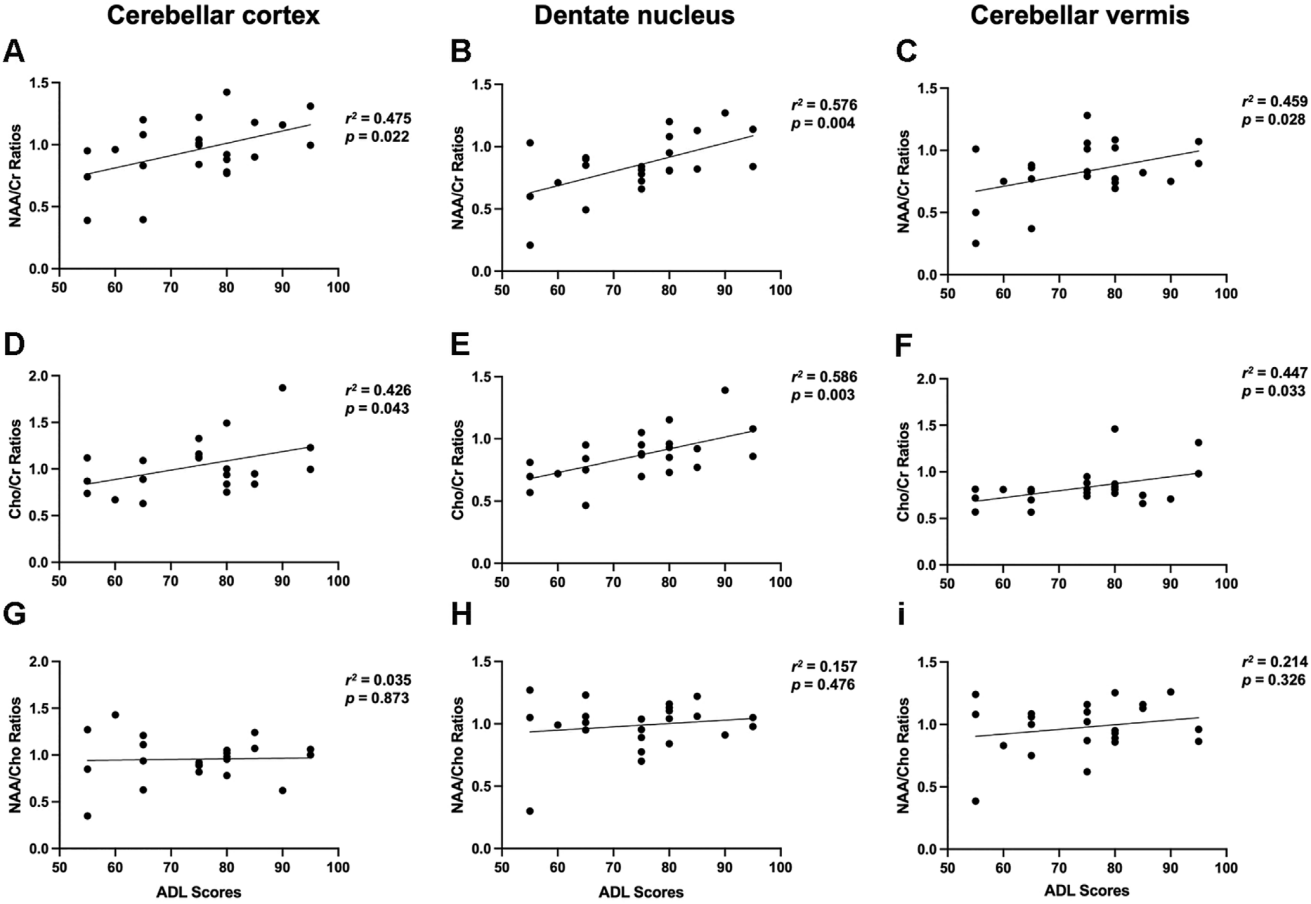
Correlations between ADL and brain metabolite ratios. In the cerebellar cortex, dentate nucleus, and cerebellar vermis: (A–C) ADL scores were significantly positively correlated with NAA/Cr ratios. (D–F) ADL scores also showed positive correlations with Cho/Cr ratios. (G–I) No significant correlations were found with NAA/Cho ratios. Pearson correlation coefficients (*r* ^2^) and *p*‐values are shown.

Subsequently, we assessed the associations of the Barthel Index scores with the metabolic ratios in the three regions of interest (Figure 4). The Barthel Index scores showed significant correlation to the NAA/Cr ratio in the cortex (*r* ^2^ = 0.475, *p* = 0.022), the dentate nucleus (*r* ^2^ = 0.576, *p* = 0.004), and the vermis (*r* ^2^ = 0.459, *p* = 0.028), which indicated that higher neuronal integrity is related to better functional outcomes. Likewise, Barthel Index scores showed significant correlations with Cho/Cr ratios in several brain regions: in the cortex (r^2^ = 0.426, *p* = 0.043), the dentate nucleus (*r* ^2^ = 0.586, *p* = 0.003), and the vermis (*r* ^2^ = 0.447, *p* = 0.033). Meanwhile, the NAA/Cho ratios were not correlated with Barthel Index scores in any region: cortex (*r* ^2^ = 0.035, *p* = 0.873), dentate nucleus (*r* ^2^ = 0.157, *p* = 0.476), and vermis (*r* ^2^ = 0.214, *p* = 0.326).

### 3.3 LASSO Analysis and Predictive Implications

**FIGURE 5:**
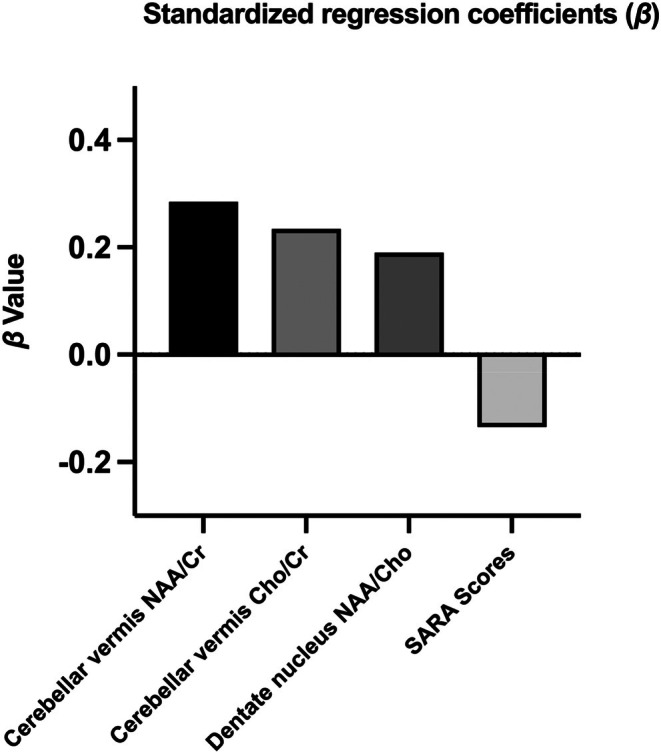
Standardized regression coefficients (*β*) from multiple linear regression. Standardized *β* values indicate that NAA/Cr and Cho/Cr ratios in the cerebellar vermis, and the NAA/Cho ratio in the dentate nucleus, positively predicted ADL scores. Conversely, SARA scores negatively predicted ADL scores. *β* values reflect the independent contribution and direction of each variable to ADL scores in the regression model.

LASSO regression was first applied to identify the most informative predictors of ADL performance among the metabolic and clinical variables. The analysis retained three predictors—Cho/Cr and NAA/Cr ratios from MRS, together with SARA score and age at onset—as relevant contributors to functional outcomes. Based on these LASSO‐selected variables, a multiple linear regression model was subsequently fitted. This model explained 45% of the variance in ADL performance (*R* ^2^ = 0.451; adjusted R^2^ = 0.329) and reached significance (*p* = 0.0229), indicating that the combined contribution of metabolic and clinical factors meaningfully accounted for functional status in SCA3 (Figure 5). Although the overall model was significant, none of the individual predictors reached statistical significance. For instance, the standardized coefficient for Cho/Cr in the vermis was 0.234 (*p* = 0.411), suggesting weak individual predictive power. This likely reflects the limited statistical power due to the small sample size (*n* = 23). Nevertheless, the overall model performance supports the potential value of integrating metabolic indices with clinical variables to predict the ability to perform ADLs in SCA3 patients.

## 4 Discussion

In this present work, we explored the association between cerebellar MRS‐obtained metabolite ratios and patients' ability to perform ADLs in individuals with SCA3. We concluded that both the NAA/Cr and Cho/Cr ratios in multiple cerebellar regions were significantly and positively associated with Barthel Index scores and that the NAA/Cho ratio was not significantly associated with Barthel Index scores. We also concluded that the association between ataxia severity (as determined by the SARA score) and ADL performance was significantly negative. Taken together, these results are suggestive of a link between local cerebellar neurochemical metabolic abnormality and lower degrees of functional independence in patients with SCA3. Relatively better neuroanatomical integrity and metabolic homeostasis in the cerebellum were related to greater independence in terms of the functional activities of daily living. Conversely, greater neuropathology was related to a greater degree of functional deterioration.

Several previous studies showed that ^1^H‐MRS‐based metabolic ratios, including NAA/Cr and Cho/Cr, can serve as imaging biomarkers of ataxic disorders and that the ratios were correlated with disease duration and clinical rating scales (Peng et al. 2019; Krahe et al. 2020; Wang et al. 2012; Wan et al. 2020; Huang et al. 2017; Lirng et al. 2012). Our results were congruent with previous research and contribute to the insight that cerebellar metabolic changes play a critical role in the clinical symptoms of SCA3.

The NAA/Cr ratio indicates relative neuronal integrity (Montes‐González et al. 2025). As a neuron‐specific metabolite, the concentration of NAA is used to be an indication of the number of neurons and their viability (Wen et al. 2017). Lower NAA is generally regarded as a marker of neuronal damage or loss (Henigsberg et al. 2017). In our study, a higher NAA/Cr ratio was associated with better ADL performance, suggesting that patients with relatively preserved cerebellar neuronal integrity exhibited higher functional capacities. This finding comports with the known pathology of SCA3, which predominantly features the degeneration of cerebellar neurons—specifically of the dentate nucleus and Purkinje cells—and is therefore secondary to worsened ataxia and decreased daily functioning (Peng et al. 2019; Chen et al. 2014; Krahe et al. 2020). Decreased NAA levels in the cerebellum and the brain stems of SCA3 patients were also documented by previous MRS‐based studies (Wang et al. 2012; Wan et al. 2020; Huang et al. 2017; Lirng et al. 2012).

Interestingly, we saw a trend between the Cho/Cr ratios and the Barthel Index scores. Choline indicates membrane phospholipid turnover and relates to cell membrane constituents, myelination, and gliosis. Elevated Cho/Cr would be associated with active membrane metabolism or glial proliferation, but reduced Cho/Cr might indicate a loss of membrane integrity or metabolic activity (Kantarci 2007; Alshammari et al. 2021). Cho levels in SCA3 patients have been inconsistently reported; however, a previous study indicated a slight reduction in cerebellar Cho/Cr ratios, which might be attributable to chronic neuronal and myelin damage (Krahe et al. 2020).

Our observation that a higher Cho/Cr ratio correlated with better ADL performance could reflect preserved membrane metabolism and a compensatory gliosis at the early or mild stages of SCA3. Furthermore, the observed decrease in Cho/Cr during the course of neurodegeneration may indicate progressive loss of glial support and cell membrane integrity. Such a finding would be in line with studies that reported reduced Cho peaks and increased Cr content in SCA3, thereby indicating relative choline dilution in the context of neuronal degeneration and compensatory creatine elevations (Krahe et al. 2020; Xing et al. 2017; Chen et al. 2022).

The LASSO regression analysis indicated that some of the metabolic variables may have the potential to predict functional outcomes (i.e., the NAA/Cr15 and Cho/Cr15 retained in the model had positive standardized coefficients—0.285 and 0.234, respectively—as shown in Figure 5). However, given the limited sample size, none of the individual predictors reached statistical significance; thus, these findings should be interpreted with caution. Although there may be multicollinearity among the predictors, the fact that some of the variables were retained in the regularized regression framework may indicate a possible relationship with the functional outcome. This preliminary observation raises the hypothesis that metabolic markers may not only reflect current ADL status but may also predict early stages of functional decline. Larger prospective studies with sufficient statistical power are needed to validate these findings and elucidate the prognostic value of metabolic markers in patients with SCA3.

### 4.1 Limitations

The multiple linear regression and LASSO analyses showed that cerebellar metabolic markers explained part of the variance in ADL performance (*R* ^2^ = 0.451), thereby suggesting that impaired cerebellar metabolism is associated with reduced functional capacity in SCA3 patients. However, our relatively small sample size (*n* = 23) significantly reduced the statistical efficacy and increased the risk of overfitting, especially in the multivariate and LASSO regression models. This limitation may have resulted in a lack of statistical significance for individual predictors. Therefore, the results of this study should be considered exploratory.

In addition, the strong correlation between SARA scores and Barthel Index scores may have masked the independent effects of the MRS‐related variables. Moreover, the relatively large voxel size at 1.5 T may have led to partial volume effects, especially in small structures such as the dentate nucleus, where signal contamination from neighboring tissue could reduce the accuracy and reliability of metabolite quantification. Future studies with higher magnetic field strengths (e.g., 3 T or 7 T MRI) and smaller voxel sizes are required to improve measurement accuracy in these regions.

## 5 Conclusions

In summary, cerebellar NAA/Cr and Cho/Cr ratios may serve as candidate neuroimaging markers of neuronal integrity and daily functioning in patients with SCA3. Exploratory LASSO analysis further suggested that their combination could have predictive value for ADL performance. Validation in larger prospective studies with advanced MRS techniques is required.

## Author Contributions

Mei Ye designed the work. Mei Ye, Xiao Ping Qiu, Shengnan Zhang, Ling Wang, Zhongming Gao, Mengyu Liu, Yaping Feng initiated the project. Mei Ye, Xiao Ping Qiu, Shengnan Zhang, Ling Wang, Zhongming Gao, Mengyu Liu, Yaping Feng collected and analyzed the data. Mei Ye wrote the manuscript. Yaping Feng commented and revised the manuscript. Mei Ye supervised all aspects of the project. All authors read and approved the final manuscript.

## Funding

This work was supported by the Medical and Health Science and Technology Project of Zhejiang Province, 2022KY261.

## Conflicts of Interest

The authors declare no conflicts of interest.

## Supporting information


**Table S1:** Comparison of Cerebellar Metabolite Ratios in SCA3 Patients Between Our Study and Two Previous Publications.

## Data Availability

The data that support the findings of this study are available from the corresponding author upon reasonable request.
