# Supplementary material for: Association Between Cerebellar Metabolic Markers and Activities of Daily Living in Patients With Spinocerebellar Ataxia Type 3
Source: Mol Genet Genomic Med. 2026 Apr 15;14(4):e70197. doi: 10.1002/mgg3.70197 (PMC13080496; doi:10.1002/mgg3.70197)
Supplement: Supplementary file 1 — Table S1: Comparison of Cerebellar Metabolite Ratios in SCA3 Patients Between Our Study and Two Previous Publications. [file MGG3-14-e70197-s001.docx]

| **Supplementary table 1. Comparison of Cerebellar Metabolite Ratios in SCA3 Patients Between Our Study and Two Previous Publications** | | | | | | |
| --- | --- | --- | --- | --- | --- | --- |
| **Regions of interest** | **Metabolic ratios** | **Our Study** | **Previous Study 1 (Chen et al., 2024)** | | **Previous Study 2 (Peng et al., 2019)** | |
|  |  | **SCA3** | **SCA3** | **Healthy Control** | **SCA3** | **Healthy Control** |
| **Cerebellar Cortex** | **NAA/Cr** | 0.94 ± 0.24 | 0.93±0.24 | 1.18±0.14 | 1.02 ± 0.27 | 1.17 ± 0.33 |
|  | **Cho/Cr** | 1.02 ± 0.27 | 1.01±0.27 | 1.11±0.23 | 0.88 ± 0.23 | 0.92 ± 0.24 |
|  | **NAA/Cho** | 0.95 ± 0.24 | 0.95±0.24 | 1.09±0.19 | 1.23 ± 0.37 | 1.31 ± 0.34 |
| **Cerebellar Vermis** | **NAA/Cr** | 0.84 ± 0.24 | 0.76±0.18 | 1.03±0.31 | 0.96 ± 0.25 | 1.22 ± 0.23 |
|  | **Cho/Cr** | 0.86 ± 0.19 | 0.91±0.16 | 0.97±0.21 | 0.88 ± 0.23 | 1.00 ± 0.18 |
|  | **NAA/Cho** | 0.98 ± 0.21 | 0.84±0.18 | 1.05±0.17 | 1.12 ± 0.28 | 1.24 ± 0.23 |
| **Dentate Nucleus** | **NAA/Cr** | 0.82 ± 0.24 | 0.90±0.27 | 1.52±0.80 | 1.07 ± 0.22 | 1.32 ± 0.24 |
|  | **Cho/Cr** | 0.81 ± 0.15 | 0.96±0.31 | 1.13±0.40 | 1.01 ± 0.20 | 1.07 ± 0.18 |
|  | **NAA/Cho** | 0.96 ± 0.21 | 0.96±0.22 | 1.30±0.19 | 1.07 ± 0.19 | 1.25 ± 0.20 |
| **Abbreviations:** **NAA**, N-acetylaspartate; **Cho**, Choline; **Cr**, Creatine. | | | | | | |
